# Supplementary material for: Are carbon emissions trading and green financial instruments synergistic? -Comprehensive quantitative research based on content analysis
Source: PLoS One. 2024 Mar 7;19(3):e0298601. doi: 10.1371/journal.pone.0298601 (PMC10919601; doi:10.1371/journal.pone.0298601)
Supplement: S1 Data — (ZIP) [file pone.0298601.s001.zip › Data/地级市Z_F工作报告.zip!/░▓╗╒/╕╖╤⌠╩╨/└┤╫╘╣1⁄2╓┌║┼í╛┬φ┐╦╩2╛▌═°í┐_2017.docx]

各位代表：

现在，我代表市人民政府，向大会报告政府工作，请予审议，并请市政协委员和其他列席人员提出意见。

一、过去六年工作回顾

本届政府任期的六年，是我市经济社会发展取得重大进展的六年。六年来，在省委、省政府和中共阜阳市委的坚强领导下，全市上下认真贯彻党的十八大和十八届三中、四中、五中、六中全会精神，深入学习习近平总书记系列重要讲话精神，科学把握大势，积极主动作为，奋力攻坚克难，较好完成了“十二五”确定的目标任务，顺利实现了“十三五”发展的良好开局。

——这六年，是综合实力大幅提升的六年。全市生产总值跨过千亿元大关，2016年达到1401.9亿元，年均增长10.1%。财政收入突破200亿元，达到226亿元，年均增长17%，总量由2010年的全省第9位上升到第6位。规模工业增加值突破500亿元，达到550.4亿元，年均增长14.5%，总量由全省第12位上升到第7位。科技创新步伐加快，高新技术企业发展到119家。粮食生产实现“十三连丰”，连续4年荣获“全国粮食生产先进市”称号。社会消费品零售总额达到759.4亿元，年均增长14.8%。

——这六年，是城乡面貌显著变化的六年。阜阳城市总体规划获省政府批准，区域性中心大城市地位进一步确立，“四位一体”发展格局初步形成。全市常住人口城镇化率达到40%，年均提高1.4个百分点。阜城建设全面提速，累计投入775亿元，建成区面积扩大到126平方公里，常住人口增加到125万。城区主干道路、桥梁、综合管网、环卫设施和园林绿化同步实施，城南新区、职教园区、阜合现代产业园区加快建设，“三项整治”成效明显，城市面貌焕然一新，对外形象大为提升，成为阜阳变化最为突出的标志。五县（市）规划建设管理不断加强，中心镇和美丽乡村建设扎实开展。

——这六年，是项目投资成果丰硕的六年。累计完成固定资产投资4668.3亿元，年均增长26.6%。重大项目建设取得新进展，金种子生态产业园、江汽中重卡制造等重点项目建成投产。省级开发园区发展到11家，面积扩大到233平方公里，太和现代医药产业入选全省首批战略性新兴产业集聚发展基地。商贸市场加快建设，颍州万达广场、临沂商城、阜阳汽贸物流园建成运营。基础设施建设步伐加快，阜新、阜六高速和阜六铁路建成通车，商阜杭高铁、郑阜高铁加快建设，市县快速通道全面建成。沙颍河航道全线复航，颍州港建成投入使用。阜阳机场年客运量达到45万人次。

——这六年，是改革开放取得突破的六年。“放管服”改革持续有效开展，累计精简行政审批事项达63.3%。工商、质监、食品药品监管体制改革全面完成。商事制度改革加快推进，各类市场主体增加到32.6万户，居全省第二位。农村综合改革稳步推进，土地承包经营权确权登记颁证顺利完成。在全省率先启动新一轮户籍制度改革，有力促进了农业转移人口市民化。财税金融改革步伐加快，政策性投融资担保体系逐步完善，8家农村信用社全部改制为农村商业银行。招商引资成效明显，累计引进市外资金5218亿元，年均增长25.5%。

——这六年，是人民生活持续改善的六年。城镇、农村常住居民人均可支配收入较快增长，分别达到25483元和9776元。民生工程不断拓展提升，累计投入563亿元。就业再就业工作成效明显，新增城镇就业39万人。持续开展“接您回家”系列活动，2.9万农民工返乡创业。城乡居民基本养老、基本医疗保险制度和大病保险、医疗救助实现全覆盖。教育设施、医疗卫生两个“三年行动计划”基本完成，新改扩建学校43所，新增医院床位1.8万张。阜城棚户区改造三年行动计划扎实推进，建成保障性住房18.5万套。城市公共交通快速发展，公共自行车系统运营良好。文化惠民工程深入实施，实现乡镇综合文化站、行政村农家书屋全覆盖。社会文明程度持续提升，22人荣获全国、全省道德模范及提名奖，169人当选中国好人、安徽好人，高思杰被中宣部授予“时代楷模”。八里河景区成为皖北首个国家AAAAA级旅游景区。

——这六年，是社会治理不断创新的六年。法治阜阳建设加快推进，网格化新型社区管理模式全面推行。信访工作形势逐步向好，社会矛盾排查预警和多元化解机制不断完善。“六五”普法任务圆满完成，“七五”普法顺利启动。食品、药品和生产安全形势持续好转，连续8年未发生重特大安全事故。平安阜阳建设深入推进，立体化数字化社会治安防控体系基本形成，禁毒工作成效明显，人民群众安全感和满意度持续提升。强武阜阳建设扎实开展，武警、人防和民兵预备役工作取得积极进展。

各位代表！刚刚过去的2016年是“十三五”开局之年，我们认真贯彻落实省委、省政府和市委决策部署，扎实做好稳增长、促改革、调结构、惠民生、防风险各项工作，较好完成了市四届人大八次会议确定的目标任务。一年来，主要做了以下工作：

一是全力促进经济增长。认真落实促进经济持续较快发展的政策措施，主要经济指标在全省继续保持总量位次前移、增速持续领先的良好态势。全市生产总值增长9%，高于全省平均水平0.3个百分点。固定资产投资增长28.6%，战略性新兴产业产值增长33.8%，高新技术产业增加值增长64.7%，均居全省第一。财政收入增长13%，规模工业增加值增长10%，均居全省第二。工业发展势头向好，新建亿元以上工业项目84个，完成投资90.6亿元。新增规模工业企业293家，居全省首位。现代农业提质增效，土地流转率提高到60%，规模养殖比重达78%。农民专业合作社和家庭农场分别达到11245家、9607家，农产品加工业产值872.8亿元，增长9.7%。现代服务业蓬勃发展，合肥百大开业运营，颍泉万达广场已经建成。电商主体发展到8300家，交易额突破80亿元，快递服务业业务收入增长62%。银行保险业稳健运行，金融机构贷款余额1571.7亿元，增长32.2%。全年引进市外投资项目1203个，实际到位资金增长22.1%。

二是全面加快城乡建设。大美阜城建设力度加大，完成投资275亿元，在全省仅次于合肥市，创历史最高水平。道路、桥梁等重点工程加快建设，全年新建城区道路118.6公里、改造54.8公里，西南大外环、阜王路南延工程、东环路及颍河大桥建成通车。市规划展示馆、市民中心主体工程完工。新建七渔河公园等城市游园10个，新增绿地1396公顷。征迁工作取得突破性进展，完成征迁965万平方米，是上年的2倍。深入推进“三项整治”，有效实施主次干道立面整治和亮化工程，扎实开展拆违拆旧整治环境专项行动。阜城数字化城管平台建成运行。成功承办了省第七届花卉博览会和第四届全民健身运动会。五县（市）城区建设全面推进，70个美丽乡村示范村建设和74个乡镇政府驻地建成区整治工作进展顺利。

三是深入推进改革创新。供给侧结构性改革“1+7”制度体系加快建立，“三去一降一补”成效明显，涉企收费减少近15亿元。深入实施“放管服”改革，建立“3+2”清单制度管理体系，全面推行公共服务清单和行政权力中介服务清单，市网上办事大厅建成运行。金融改革稳步推进，累计发放“4321政银担”贷款72.7亿元，新增“新三板”和省股权交易中心挂牌企业150家，均居全省首位。科技创新成效明显，新增高新技术企业38家，发明专利授权量517件。

四是扎实开展脱贫攻坚。认真贯彻落实精准扶贫、精准脱贫方略，全面实施脱贫攻坚“十大工程”。突出产业脱贫，发展特色种养扶贫基地698个，建成村光伏电站211个、户用光伏电站1.08万个，51个村列入首批国家级旅游扶贫重点村。加强就业脱贫，建成村级扶贫车间512个。启动易地扶贫搬迁，完成3610人搬迁和8049户危房改造。推进健康脱贫，全面落实“两降四提一兜底”政策。大力实施水电路“三项工程”，建成农村饮水安全工程56处，解决了95万人饮水安全问题。农村电网改造完成投资15.6亿元，投资额连续两年居全省首位。完成农村道路畅通工程3533公里。脱贫攻坚首战告捷，实现17.8万人脱贫、169个村出列，超额完成年度任务。

五是切实改善民计民生。大力推进基本公共服务均等化，33项民生工程全面完成。高度重视就业创业，全年新增城镇就业4.9万人、农村转移劳动力11.6万人。建立机关事业单位工作人员养老保险制度，调整提高企业和机关事业单位退休人员基本养老金，城乡低保月人均补差分别增长17.2%和52.4%。大力促进义务教育均衡发展，新建公办幼儿园45所、竣工26所，教育教学质量得到提升。市人民医院新区建成使用，安医大附属阜阳医院即将开诊。全面二孩政策得到落实，人口自然增长率8.5‰。生态建设持续加强，森林增长工程全面完成，颍州西湖国家湿地公园通过验收。深入实施水环境综合整治和大气污染防治“颍淮蓝天工程”，秸秆禁烧和综合利用工作成效显著。妇女、儿童及老龄、老干部工作进一步加强。民族、宗教、统计、档案、地方志、地震、外事、侨务、台湾事务等工作也都取得了新的成绩。

六是大力加强自身建设。扎实开展“两学一做”学习教育和“讲看齐、见行动”学习讨论，政府系统党的建设得到加强。深入推进依法行政，自觉接受人大法律监督和政协民主监督，全年办理人大代表建议124件、政协委员提案215件。首批两个地方性法规《阜阳市地下水保护条例》、《阜阳市城市绿化条例》颁布实施，首部政府规章《阜阳市户外广告和招牌设置管理办法》公布施行。切实加强党风廉政建设，强化行政监察和审计监督，认真落实巡视反馈问题整改任务，加强政务督查和目标管理绩效考核，政府执行落实能力进一步提升。

各位代表！过去的六年，是阜阳发展史上综合实力提升最快、城乡面貌变化最大、人民群众得到实惠最多的六年。成绩来之不易，必须倍加珍惜。这是省委、省政府和市委正确领导的结果，是市人大、市政协大力支持的结果，是全市上下凝心聚力、锐意进取、扎实苦干的结果。在此，我代表市人民政府，向全市人民，向各民主党派、各人民团体和社会各界人士，向驻阜解放军指战员、武警官兵和政法干警，向所有关心支持阜阳发展的同志们、朋友们，表示衷心的感谢！

在肯定成绩的同时，我们也清醒地看到，我市经济社会发展中仍存在一些困难和问题。一是发展不足依然是面临的最大矛盾，经济总量不大，人均水平偏低，工业化、城镇化水平不高。外贸进出口出现下滑，民间投资增幅回落。二是脱贫攻坚任务艰巨，全市贫困人口较多、致贫原因复杂、产业基础薄弱，如期实现脱贫目标还需付出艰辛努力。三是补齐短板的压力较大，水、电、路等基础设施建设相对滞后，教育、医疗、养老等领域供需矛盾较为突出。四是维护社会和谐稳定任重道远，食品、药品和生产安全监管形势严峻，环境保护、社会治理等方面还存在不少问题。同时，一些干部干事创业、开拓创新、争先创优的意识有待增强，引领发展、破解难题、担当作为的能力有待提升，尤其是慢作为、不作为的现象还不同程度地存在，党风廉政建设和反腐败斗争形势依然严峻复杂。对此，我们一定高度重视，下大力气认真解决这些问题，绝不辜负人民的期待。

二、未来五年工作重点

未来五年,是全面建成小康社会的决胜阶段,是我市必须大有作为而且能够有大作为的黄金发展期。我们既有人力资源、市场潜力、交通区位等比较优势，又有国家促进中部崛起、中原经济区和淮河生态经济带建设、省委省政府加快皖北振兴等政策机遇。同时，经过多年努力，全市政治生态风清气正、经济发展势头强劲、社会大局和谐稳定、干部队伍精神饱满。只要我们乘势而上，团结拼搏，就一定能够开创经济社会发展的崭新局面！

经济社会发展的指导思想是：全面贯彻党的十八大和十八届三中、四中、五中、六中全会精神,深入学习贯彻习近平总书记系列重要讲话精神,认真落实省第十次党代会和市第五次党代会决策部署，以新发展理念统领发展全局,以推进供给侧结构性改革为主线，坚持“双轮驱动”发展战略，大力实施五大发展行动计划，坚决打赢脱贫攻坚战,确保如期实现全面小康目标，奋力走在皖北振兴前列，成为安徽发展重要增长极。

未来五年，经济社会发展的主要目标是：

——综合实力明显增强。主要经济指标总量和年均增速领先皖北,增长速度高于全省。到2021年末,全市生产总值超过2300亿元,财政收入力争突破400亿元。三次产业结构更趋合理，基本建成特色鲜明的现代产业体系。

——城市能级大幅提升。新型城镇化步伐不断加快，中心城市规模快速扩张、功能明显增强、综合承载力大幅提升，常住人口城镇化率达到49%，成为区域性商贸中心、教育中心、文旅中心、医疗中心和综合交通枢纽。

——改革创新实现突破。重点领域和关键环节改革取得决定性成果,开放型经济新体制基本形成。自主创新能力显著增强,科技进步对经济增长贡献率明显提高。加快推进人力资源大市向人力资本大市转变，努力打造制造强市、科教大市和技工大市。

——生态环境持续优化。生产生活方式加快向绿色、循环、低碳转变,自然生态资源得到有效保护和合理开发，水、大气、土壤等污染有效整治, 环境质量进一步改善，如期实现省下达的节能减排目标。

——人民生活更加幸福。城镇、农村常住居民人均可支配收入增幅高于全省平均水平,基本公共服务追赶全省平均水平。社会文明程度、公民文明与健康素质明显提升。群众权益得到充分尊重和切实保障。

——制度体系不断健全。法治阜阳、诚信阜阳、平安阜阳建设扎实推进。人民民主不断扩大,法治政府建设步伐加快,依法治市全面推进,司法公信力明显提高。

围绕上述目标，加快推进五大发展行动计划实施方案。

（一）加快推进创新发展，切实壮大实体经济规模

创新是引领发展的第一动力。坚持把创新摆在缩小差距、加速追赶的核心位置，大力实施“双轮驱动”发展实体经济三年行动计划，促进“三产融合”、“四化同步”。

扎实推进产业创新，着力增强整体经济实力。实施工业发展三年行动计划，统筹推进新兴产业培育和传统产业改造升级，重点发展现代医药、绿色食品、煤电化工、节能环保、装备制造、新材料、新一代信息技术等七大主导产业，加快形成特色工业产业集群，努力打造千亿元产业、百亿元企业。实施现代生态农业产业化三年行动计划，积极构建现代生态农业产业化发展“三圈”模式，坚持以市场为导向，通过品牌引领、循环利用和“三产融合”，以品牌化运营打造产品生态圈，以联合体组织打造企业生态圈，以复合式循环打造区域产业生态圈。调整优化产品、生产、产业结构，实施“绿色食品产业工程”，培育龙头企业“甲级队”，促进“百亿粮仓”向“千亿厨房”转变。加快服务业发展提速提质，高水平建设一批现代服务业集聚区。实施电商阜阳建设工程，促进互联网产业发展。实施旅游业发展三年行动计划，推进核心景区培育工程和乡村旅游提升工程，加快建设旅游强市。

扎实推进科技创新，着力培育发展内生动力。按照省政府提出的构建技术创新支撑体系、全链条跟进的创新平台支撑体系、全生命周期的创新金融支撑体系、全要素服务的创新政策支撑体系的要求，系统推进技术、平台、金融、政策四大创新支撑体系建设，着力塑造更多依靠创新驱动的引领型发展。切实加强与高校、科研机构的合作，加大科技成果转化力度，完善政产学研用结合机制，激发全社会创新活力和创造潜能。大力促进互联网、云计算、大数据、物联网与现代制造业结合，壮大一批骨干企业。实施质量品牌升级工程，广泛开展“增品种、提品质、创品牌”专项行动，推进质量强市建设。

扎实推进体制创新，着力激发经济发展活力。以解决发展中的突出问题和结构性矛盾为导向,推动重点领域和关键环节改革攻坚。深化行政管理体制改革，加快政府职能转变，提升政府服务管理效能。加大国资国企改革力度，健全现代企业制度，积极开展混合所有制改革。深化投融资体制改革，完善金融更好服务实体经济和产业创新的链条。推进以农村集体产权制度为重点的农村综合改革，为农村持续稳定发展提供制度保障。

（二）加快推进协调发展，着力构建区域联动格局

协调是持续健康发展的内在要求。坚持在协调推进中拓展发展空间，在厚植优势中激发动力，在补齐短板中增强后劲。

加快区域协同联动发展。全面融入长三角、中原经济区、淮河生态经济带，积极促进区域空间布局优化、生态环境共治、基础设施共享，提升参与区域合作分工的能力，加快打造国家中部地区重要的综合交通枢纽、中原地区重要新兴制造业基地和能源基地、皖豫省际区域性中心城市。

加快现代化大城市建设。坚持“一个尊重五个统筹”，立足建设富有竞争力的现代化大城市，实施城市建设三年行动计划，集中项目、要素、资金，主攻城南新区，开发高铁新区和西湖新区，统筹推进颍州、颍泉、颍东片区建设，进一步拉大城市框架，促进市区一体化，提升中心城市能级。加快完善城市路网、综合管廊、园林绿化、信息通讯和公共停车场等基础设施，推进智慧城市、海绵城市和信息惠民城市建设。积极创建全国绿化模范城市、国家园林城市和省环保模范城市、省卫生城市，为争创全国文明城市奠定基础。

加快城乡发展一体化。坚持以工促农、以城带乡，促进城乡在规划布局、产业发展、要素配置、基础设施、公共服务、生态环保等方面相互融合、共同发展。实施县域经济振兴工程，支持临泉县重点发展劳务经济、打造人力资本大县，太和县重点发展现代医药产业、打造医药高地和诗画之乡，阜南县重点发展农产品加工业、打造家居家俬生产基地，颍上县重点发展全域旅游、打造旅游文化强县，界首市重点发展节能环保产业、打造双创高地和品质慢城。有效推进特色城镇、美丽乡村建设，持续深入开展“三线三边”环境治理，推动移风易俗，树立文明乡风，建设幸福生活美好家园。

（三）加快推进绿色发展，大力加强生态文明建设

绿色是永续发展的必要条件。始终坚持绿色发展战略，使阜阳天更蓝、地更绿、水更清、生态更安全。

着力构建绿色生态屏障。落实主体功能区规划,逐步建立科学合理的城镇化格局、农业发展格局和生态安全格局。坚持人水和谐、绿色共享，扎实推进泉河生态经济示范区、沙颍河生态景观带、颍州西湖生态湿地保护区和旅游度假区建设。统筹生态廊道构建、绿色产业发展、绿色村镇建设，优化生产、生活、生态空间布局，筑牢生态安全屏障。

严守资源环境生态红线。健全大气、水、土壤污染防治机制，严格执行生态环境损害责任追究制度。强化约束性指标管理，落实能源、水资源消耗、建设用地总量和强度双控行动。启动实施综合治水三年行动计划，全面推行“河长制”。建立排污权、碳排放权、用能用水权交易制度，完善生态保护补偿机制。

大力发展绿色循环低碳经济。强化全民生态文明意识，推行绿色低碳生产生活方式，推进国家循环经济示范城市建设, 构建复合联动的生态产业体系，提高资源节约集约利用水平，让尊重自然、呵护生态成为全市人民的自觉追求。

（四）加快推进开放发展，不断拓展自主增长空间

开放是繁荣发展的必由之路。深入实施开放引领战略,着力拓展开放空间，提升开放层次,推动阜阳大发展、快发展。

构建开放发展大通道。实施综合交通建设三年行动计划，全面推进铁路、公路、航道、航空、电网、油气管道、信息网络、商务平台建设，打造现代综合交通运输体系、能源体系、网络信息体系和物流体系，努力补齐基础设施短板。加强多种运输方式衔接，加快形成高铁、普铁双“米”字型格局，实施阜阳机场改造升级工程，谋划建设临空物流园，进一步提升综合交通枢纽地位。

建设开放合作新平台。以增强综合承载能力为目标，以夯实基础、完善配套、优化服务、合作共建为手段，实施园区转型升级三年行动计划，推动工业开发园区、商贸物流园区、现代农业园区、“双乡”“双创”园区、职教园区五大提升工程，努力形成主业突出、特色鲜明、错位发展的新格局。积极申报国家铁路、航空一类口岸，发展壮大口岸经济。推进外贸主体培育壮大工程，建设跨境电子商务产业园。

打造招商引资“强磁场”。坚持引资与引技引智并重，制定招商引资优惠政策，完善激励奖励办法，广泛开展亲情招商、专业招商和以商招商。大力发展会展经济，推动招商引资与产业升级互动，全面提升精准招商水平，把阜阳打造成为富有吸引力的投资热土。

（五）加快推进共享发展，持续增进人民群众福祉

共享是加快发展的根本目的。牢固树立以人民为中心的发展理念，让人民群众在共建共享中有更多获得感。

坚决打赢脱贫攻坚战。以增加贫困群众收入、改善贫困地区生产生活条件为重点,认真落实省委省政府“六看六确保”和“四个严”的要求，深入实施脱贫攻坚十大工程，推动精准扶贫、监测评估、防范返贫、责任落实全覆盖，做到扶真贫、真扶贫、真脱贫，到2020年实现户户脱贫、村村出列、县县摘帽，确保全面小康路上不落下一人！

织牢织密民生保障网。持续增加城乡居民收入，努力使增速高于全省平均水平。完善公共就业创业服务体系，推动实现更充分更高质量的就业。优先发展教育，打造优质均衡的基础教育、更具特色的现代职业教育。推进健康阜阳建设，全面提升医疗卫生服务能力。加快构建更公平、全覆盖、可持续的社会保障制度，扩大公共文化服务供给，让人民群众物质更富足、精神更富有、生活更幸福。

切实筑牢公共安全网。深化平安阜阳建设，坚持预防为主、防控结合，完善公共安全防控体系，构建风险排查、研判、预警、防范、处置和责任落实机制，全面加强社会治安、安全生产、食品药品安全和信息网络安全建设，让阜阳社会安定、人民安康。

三、2017年重点工作安排

今年是党的十九大召开之年，是实施“十三五”规划的重要一年，也是供给侧结构性改革的深化之年。坚持“稳中求进、进中求快”工作总基调，经济社会发展的主要预期目标是：全市生产总值增长9%以上，财政收入增长10%以上，固定资产投资增长20%以上，规模工业增加值增长10%以上，社会消费品零售总额增长12%以上，城镇、农村常住居民人均可支配收入分别增长9%和10%以上，城镇登记失业率控制在4.5%以内，完成省下达的节能减排任务。

实现上述目标，突出抓好六项重点工作。

（一）大力促进实体经济增长

积极培育工业主导产业集群，促进现代制造业基地建设。切实扩大工业投资规模，引导更多劳动、土地、资本、技术、管理、知识、信息等要素投入到工业领域，培育壮大富有竞争力的工业主导产业集群。围绕扩大工业增量，扎实开展产业链精准招商，引进一批成长性好、带动性强、竞争优势明显的项目。着力提升太和现代医药产业集聚发展基地建设水平，培育一批市级战略性新兴产业基地。围绕扩张工业存量，大力促进绿色食品、煤电化工等传统产业转型升级，加快煤基新材料、江汽中重卡制造、秸秆综合利用环保产业园建设进度，扎实推进阜阳卷烟厂易地技改项目，力争下半年开工建设华润电厂二期工程。发展壮大中小企业，全年新增规模工业企业150家以上。围绕壮大园区平台，加强基础设施建设，开展闲置低效用地清理，提高园区发展集聚度和集约化水平，支持有条件的地方创建国家级开发区和高新技术开发区。

扎实推进现代生态农业产业化，促进农业提质增效。始终坚持重农优农，深入推进农业供给侧结构性改革，加快结构调整步伐，优化产业体系、生产体系、经营体系。大力发展专用品牌粮食，扩大高效经济作物面积，提升畜牧生态养殖和水产健康养殖水平，增加绿色农产品供给。加快培育品牌农产品，力争新认证“三品一标”农产品60个以上。培育市级以上农民合作社示范社410家、家庭农场220家以上，发展现代农业产业化联合体80个。新建高标准农田60万亩，农作物综合机械化率达到89%。积极推行“互联网+现代农业”模式，扩大农业物联网等新技术应用。大力促进农副产品加工转化，打造粮食、蔬菜、肉类等加工产业集群，力争农产品加工业产值突破1000亿元。

加快建设区域旅游目的地，促进现代服务业集聚发展。充分挖掘历史文化资源，科学编制旅游发展规划，启动颍州西湖景区建设工程。推进八里河景区、祥源颍淮文旅城建设，打造乡村旅游精品线路，发展民宿客栈、采摘篱园、特色农庄，力争旅游业年收入突破100亿元。顺应消费升级新趋势，发展医疗服务、健康养老、健身休闲等产业，推动研发设计、信息技术服务等现代服务业。发挥新消费引领作用，深入开展“十大扩消费行动”。加快传统商圈提档升级，促进实体零售创新转型。推进阜阳铁公水综合物流港建设，打造国家区域级流通节点城市。发展电商线上线下相结合的新业态，推进“电子商务进农村”全覆盖。实施外贸企业孵化工程，加强出口示范基地建设。

着力打造活力迸发的创新生态圈，促进信息化与工业化深度融合。创新科技成果转化模式，加大高端人才的培育、引进和集聚力度，实施股权和分红激励政策，催生更多创新资源，打通创新人才、核心技术到产品、产业转化的通道。加快完善创新金融服务链条，设立天使投资基金和产业投资基金。实施智慧阜阳建设工程，发挥大数据的引领作用，对实体经济进行全方位改造升级，着力发展共享经济。

（二）全面加速新型城镇化进程

加快大美阜城建设。紧紧围绕“规模大、设施全、经济活、环境优、形象美”的建设目标，推进阜城大投入、大建设、大发展。进一步完善规划体系，加快编制高铁新区、西湖新区建设规划和城南新区二期控规，谋划编制城市快速立体交通建设规划。实施道路桥梁提升行动，加快建设解放北路泉河桥、航颍路泉河桥和向阳北路、淮河路西延工程，提升改造沙河路、临泉路、河滨路，拆除重建阜裕大桥，开工建设颍州北路颍河桥。实施园林绿化提升行动，加快建设七渔河防灾避险公园和岳家湖公园、刘锜公园，改造提升青颍公园、文峰公园，打造四季常青、五彩缤纷的城市形象。实施公用事业提升行动，加快市博物馆、科技馆、图书馆、大剧院建设，开工建设奥林匹克体育公园。完善供电、供水、供气和环卫等市政设施网络，开工建设市生活垃圾焚烧发电厂二期工程。加快建设公交六场、七场，创建省级公交示范城市。加强住宅小区物业管理，破解物业监管难题。新建56个公共停车场，有效缓解城市交通拥堵压力。深入推进以“三项整治”为主要内容的市容环境综合治理，持续开展拆违拆旧、立面整治和市容保洁专项行动，打造设施齐全、环境优美、文明有序、品位高雅的示范街区。加快推进亮化提升行动，高标准建设一批重点亮化工程，形成“白天看绿化、晚上看亮化、全天看美化”的城市景观。

持续推进县乡建设。深入实施“四创一建”行动，支持五县（市）按照中等城市标准，加强规划建设管理,拉大城市框架，完善基础设施和公共服务,引进建设一批城市综合体、商贸市场和学校医院，提升县城综合承载能力。编制完善美丽乡村建设规划，完成75个乡镇政府驻地建成区整治和71个中心村建设。积极推动生活垃圾收集处理全覆盖，不断改善农村环境。

完善现代基础设施体系。大力推进安全供水、城市防洪、污水治理三大工程，积极促进引江济淮及配套工程建设，开工建设淮河干流王临段、洪河综合治理等防洪工程。实施城区40条内河综合治理，推进22条黑臭水体治理工程。大力支持商阜杭、郑阜高铁加快建设。争取阜阳—霍邱—合肥、阜阳—淮滨和颍上—阜南—临泉—界首3条高速开工，建设阜城东北大外环、南北大通道、临泉至界首快速通道。新建国省干线465公里、农村道路1887公里。开工建设阜阳复线船闸，及早建成颍上复线船闸、耿楼复线船闸。扎实推进淮河阜阳港前期工作，综合整治淮河干线航道、泉河航道。

（三）持续深化重点领域改革

深化供给侧结构性改革。认真落实“三去一降一补”任务，重点抓好调整产业结构、提升产品质量、化解过剩产能、降低企业成本和防范金融风险等工作。推进棚户区改造货币化安置和公租房货币化保障，加强住房市场监管，引导房地产回归居住功能，促进房地产业平稳健康发展。

深化行政体制改革。完善权责清单制度，加强事中事后监管，实现“双随机、一公开”全覆盖。深入推进商事制度改革，扩大“证照合一”覆盖面。全面完成城市执法体制改革，建成县城数字化城管平台。加快公共资源交易监管体制改革，推行“互联网+政务服务”，建成投资项目在线管控平台。开展“减证便民”专项行动，最大程度方便群众和企业。

深化农村综合改革。巩固扩大土地承包经营权确权登记颁证成果，建立农村产权交易平台，推进土地承包经营权、农村住房财产权抵押贷款等改革试点。开展农村集体资产清产核资，推进农村集体资产股份合作制和资源变资产、资金变股金、农民变股东“三变”改革试点步伐。全面推开“三项补贴”改革，深化供销社体制改革。完成第三次全国农业普查。

深化财税金融体制改革。理顺市、区财政事权和支出责任。安排8亿元专项资金，建立公开透明的财政支持产业发展、创新创业人才建设政策体系。加大财政支出优化整合力度，逐步将竞争性领域专项资金转化为股权投资基金。推进国税、地税征管体制改革，落实“营改增”试点政策。发展壮大农村商业银行，积极引进股份制商业银行，支持各类政策性银行和国有股份制银行提升服务能力，扩大社会融资规模，进一步提高贷存比。抢抓国家扶贫县企业上市绿色通道政策机遇，积极促进本土企业上市，综合运用多样化债权融资工具，提高直接融资比重。完善政策性融资担保体系，健全融资担保风险补偿机制，积极拓展特色保险品种。

大力发展民营经济。全面落实支持民营经济发展各项政策，放宽放活非公有制经济市场准入。适度加大PPP模式推广运用力度，促进民间投资企稳回升。依法保护企业家财产权和创新收益，着力构建“亲”“清”新型政商关系，营造尊重、支持、服务、宽容企业家的环境，让企业家专注创业、放心创业、愉快创业！

（四）坚决打赢脱贫攻坚年度战役

构建精准扶贫保障机制。深入实施脱贫攻坚十大工程，大力发展“龙头企业+合作社+贫困户”生产经营模式。新建村级光伏电站207个、户用1万个，建设20个乡村旅游扶贫重点村。实施贫困人口易地搬迁4323人，建成村级就业扶贫车间1180个。持续推进水、电、路“三项工程”。严格执行农村低保标准和扶贫标准“两线合一”，全面落实贫困人口“三保障一兜底”综合医保政策，确保完成19.2万人脱贫、170个村出列的年度任务。

构建督查考评保障机制。认真落实脱贫攻坚工作成效考核办法，坚持脱贫攻坚十大工程调度联络机制。积极配合第三方监测评估，进一步完善督查巡查、走访暗访制度，对拟出列的贫困村、脱贫的贫困户巡查全覆盖，确保脱贫精准度和实效性。

构建防范返贫保障机制。建立返贫预警制度，加强返贫信息管理，通过调查、评议、审核、认定等途径确认返贫户，及时衔接帮扶机制。对已脱贫的村、户，一定时期内继续保留原有扶贫政策。为贫困户量身定制保险产品，夯实稳定脱贫基础。

构建责任落实保障机制。完善脱贫攻坚责任体系，健全“单位包村、干部包户”和驻村扶贫工作队管理办法，建立考核问责机制，形成责任链闭环运行。深入开展“百企帮百村”精准扶贫行动和扶贫志愿者行动，凝聚脱贫攻坚强大合力。

（五）尽心尽力保障改善民生

加强民生保障和就业工作。按照省政府统一部署，组织实施33项民生工程。坚持建管并重，保障资金配套，加快实施进度，提升建后管养、绩效管理和政务公开水平，不断推动民生工程提质增效。大力促进农村劳动力、高校毕业生、退役军人和城镇就业困难人员等重点群体就业，确保零就业家庭动态“清零”。深化“接您回家”系列活动，完善扶持政策，推动返乡下乡创业就业。配合做好工伤保险基金省级统筹工作，规范基本医疗、失业和生育保险市级统筹。完善城乡最低生活保障和社会救助制度。新增保障性安居工程59228套，基本建成27819套，完成8398户农村危房改造任务。

统筹发展社会事业。继续改善基础教育办学条件，加快阜阳一中、阜阳七中、阜阳十中和实验中学、成效中学新校区建设，着力提升教学水平，确保所有县市区通过义务教育均衡发展国家认定。实施第三期学前教育行动计划。支持阜阳师范学院更名为大学。加快推进阜阳职业技术学院新区建设，拓展本科教育。开工建设阜阳幼儿师范高等专科学校和阜阳技师学院二期工程。加快与国内外知名高校、科研机构合作进程，提高阜阳职教园区建设水平，努力创建开放式大学。深化医药卫生体制综合改革，推进医联体建设，促进分级诊疗，不断提高县域就诊率。改革完善计划生育服务管理，加强妇产科、儿科建设。加快建设市人民医院新区二期、市中心血站、中铁阜阳中心医院二期等项目，促进市五院新区、市六院新区、阜阳民生医院及早建成使用。加强文物和文化遗产保护利用，建设一批文化小镇、文化街区。培育和践行社会主义核心价值观，加强社会公德、职业道德、家庭美德和个人品德建设，传承优良家风家训。进一步弘扬主旋律，传播正能量，提升阜阳新形象。切实维护妇女儿童权益，健全农村留守儿童和老年人关爱帮扶体系。发展老龄事业和社会福利事业，保障残疾人合法权益。

全面加强生态环境保护。持续抓好重点领域污染治理，深入开展工业废气、城市扬尘、餐饮油烟、机动车尾气专项整治和燃煤锅炉改造，淘汰所有黄标车。加强秸秆禁烧和综合利用，培育一批秸秆资源综合利用龙头企业，支持秸秆收储体系建设。用足用活“增减挂钩”政策，稳步推进耕地数量、质量、生态“三位一体”保护，争创国家节约集约用地模范县（市）。大力推进林业增绿增效行动，成片造林5万亩，建设森林长廊示范段100公里，创建省级森林城镇8个。

（六）全力维护社会和谐稳定

筑牢安全生产防线。严格落实安全生产责任制，深化“铸安”行动，全面排查整治各类事故隐患，突出抓好消防安全、道路交通、危险化学品、建筑施工等重点领域安全防范，强化安全监管执法，坚决杜绝重特大事故、遏制较大事故、减少一般事故。

保障群众食品药品安全。按照“四个最严”的要求，突出重点领域、重点地区，全面实施食品药品风险分级管理，构筑事前预防、事中事后监管的责任网，加强“农田到餐桌”的全过程监管，积极创建省级食品药品安全示范城市和农产品质量安全县。

加强和创新社会治理。深化信访工作制度改革，实施化解信访积案专项工程。加强应急平台和专业队伍建设,提高应急处置保障能力。持续开展“守护平安”系列行动，依法严厉打击各类违法犯罪行为,深入开展防范打击非法集资、电信网络诈骗犯罪专项行动,进一步提升人民群众安全感和满意度。推进社会诚信体系建设，营造优良信用环境。加强国防动员、后备力量建设和人民防空工作,巩固军政军民团结,积极争创全国双拥模范城。高度重视国家安全、保密工作。认真做好气象、地震等工作。继续做好统计、档案、地名、地方志和民族、宗教、外事、侨务、台湾事务等工作。

各位代表，切实做好今年各项重点工作，确保完成全年目标任务，必须始终坚持经济工作项目化、项目工作责任化，加快完善项目推进四项机制。一是完善项目谋划编制机制。瞄准政策导向，立足资源禀赋，围绕投资需求，重点谋划编制一批打基础、管当前、利长远的重大项目，做实做细项目编制每一个环节，加强项目申报推介工作，形成滚动增加、梯次推进的良性循环。二是完善项目投资融资机制。充分发挥市城投、交投、城南投资公司等政府投融资平台作用，提高运营效益，带动社会投入。启动实施金融服务实体经济工程，有效撬动融资杠杆。加大招商引资工作力度，筹建“阜阳之友联谊会”。三是完善项目建设管理机制。坚持政府引导、市场运作、建管结合、一体推进，优化征地拆迁机制、招标投标机制和运营管理机制，做到精准对接、规范透明、高效廉洁，全面提升项目建设管理水平。四是完善项目调度推进机制。围绕896个市级重大项目，认真落实市县领导联系重点项目制度和“四督四保”推进机制，筹建基建类、产业类两个市级项目调度指挥中心，推行项目信息化台账管理和动态管控，实行每月一调度、每季一现场、半年一观摩、每年一奖惩，力促谋划项目早开工、在建项目早建成、建成项目早见效，推动项目建设提速提质提效，为经济社会持续较快发展提供有力支撑。

四、全面提升政府工作水平

实现未来五年宏伟目标，确保完成今年各项任务，对政府工作提出了新的更高的要求。各级政府及其工作人员务必进一步加强自身建设，切实履职尽责，不断提升施政水平。

（一）强化政治定力，建设学习型政府。切实把学习作为提升素质、增强本领、推动工作的重要途径，建立健全抓学习、促学习的责任机制，大力推进学习型机关建设。强化理论武装，坚定理想信念，牢固树立“四个意识”特别是核心意识、看齐意识，坚定自觉地向党中央看齐、向党的理论和路线方针政策看齐、向党中央的决策部署看齐。坚持与时俱进，积极顺应和把握经济发展新常态，着力打造高素质的公务员队伍，切实增强研判形势、驾驭全局、推进发展的能力。

（二）严格依法行政，建设法治型政府。坚持法定职责必须为、法无授权不可为，严格依照法定权限和程序履行职能，自觉运用法治思维和法治方式推动工作。完善政府立法机制，推进重大事项合法性审查全覆盖。规范政府决策行为，健全决策咨询、政府法律顾问等制度，推进决策科学化、民主化、规范化。主动接受人大依法监督和政协民主监督，严格执行市人大及其常委会的决议、决定，认真办理人大代表建议和政协委员提案，广泛听取各民主党派、工商联、无党派人士意见，充分发挥工会、共青团、妇联等人民团体的作用，自觉接受司法监督、社会监督和舆论监督，让权力在阳光下运行。

（三）加快职能转变，建设服务型政府。坚持把公共服务和社会管理摆在更加重要的位置，把促进社会公平正义、增进人民福祉作为出发点和落脚点，完善领导干部直接联系和服务群众机制，深入开展走访基层群众活动，多渠道了解社情民意、回应群众关切，多谋民生之利，多解民生之忧。进一步加大经济环境治理力度，努力营造良好的发展环境。全面加强政务公开，加快构建面向公众、开放共享的一体化网上政务服务平台，畅通联系服务群众的“最后一公里”。

（四）切实改进作风，建设效能型政府。坚持把抓落实作为政府工作的生命线，持续深化“严纪律、转作风、提效能、促服务”活动，大力倡导说干就干、干就干好，多划句号、少划逗号，提高一次部署完成率。树立崇尚实干、鼓励创新的工作导向，弘扬“五干”作风、争当“五型”干部。强化政务督查，严格行政问责，确保政令畅通、令行禁止。建立健全正向激励和容错纠错机制，为敢闯敢试者撑腰，为创新创业者鼓劲，让广大干部想干事、会干事、敢干事、不出事、干成事。

（五）坚持从严治政，建设廉洁型政府。切实履行全面从严治党主体责任，巩固拓展“两学一做”学习教育成果，持续提升政府系统从严管党治党水平。认真执行《准则》和《条例》，严格落实中央八项规定精神和省委30条、市委36条要求，驰而不息推进作风建设，严厉查处发生在群众身边的“四风”和腐败问题。厉行勤俭节约，压缩一般性支出，做到“三公”经费只减不增。全面加强反腐倡廉制度建设，健全廉政风险防控机制，推进审计监督全覆盖，着力营造政治上的绿水青山。

各位代表！新蓝图描绘新愿景，新起点开启新征程。让我们在中共阜阳市委的坚强领导下，牢记使命，勇于担当，撸起袖子加油干，甩开膀子使劲干，努力开创全市经济社会发展新局面，以优异的成绩迎接党的十九大胜利召开！
